# Supplementary material for: Improved GPCR ligands from nanobody tethering
Source: Nat Commun. 2020 Apr 29;11:2087. doi: 10.1038/s41467-020-15884-8 (PMC7190724; doi:10.1038/s41467-020-15884-8)
Supplement: Supplementary file 1 — Supplementary Information [file 41467_2020_15884_MOESM1_ESM.pdf]

SUPPLEMENTARY INFORMATION

Cheloha *et al.* Improved GPCR ligands from nanobody tethering

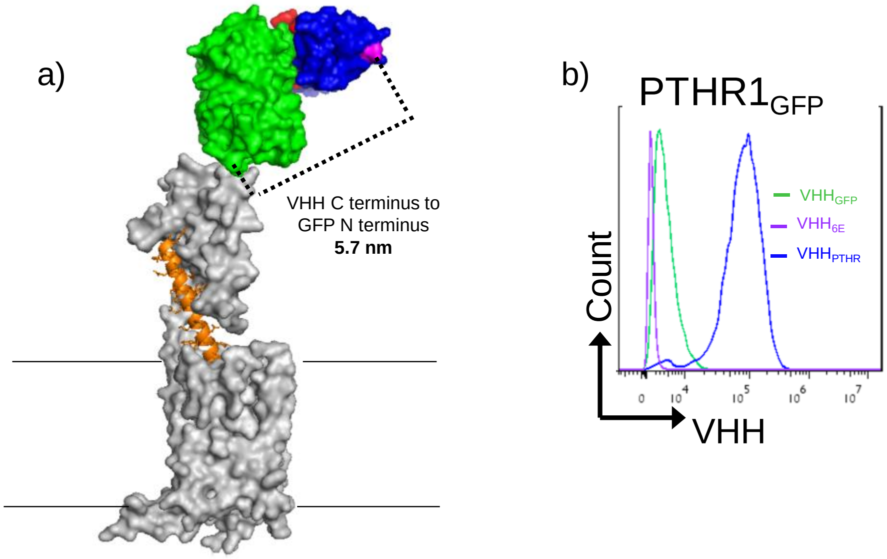

c)

Peptide or conjugate	PTHR1$_{GFP}$
PTH(1-34)	0.4 ± 0.8
PTH(1-14)	2.3 ± 1.2
PTH(1-11)	79 ± 45
PTH(1-10)	2552 ± 653
PTH(1-9)	Inactive at 10,000 nM
VHH$_{PTHR}$-PTH(1-14)	0.5 ± 0.2
VHH$_{PTHR}$-PTH(1-11)	0.5 ± 0.1
VHH$_{PTHR}$-PTH(1-10)	ND
VHH$_{6E}$-PTH(1-14)	ND
VHH$_{6E}$-PTH(1-11)	Inactive at 100 nM
VHH$_{6E}$-PTH(1-10)	Inactive at 100 nM
VHH$_{6E}$-PTH(1-9)	ND
VHH$_{GFP}$-PTH(1-14)	1.8 ± 0.6
VHH$_{GFP}$-PTH(1-11)	8.0 ± 3.4
VHH$_{GFP}$-PTH(1-10)	Inactive at 100 nM
VHH$_{GFP}$-PTH(1-10)	Inactive at 100 nM

Supplementary Figure 1. Use of PTHR1-GFP. a) Hypothetical structure of PTHR1 with GFP engrafted into exon 2 bound to a GFP binding nanobody. b) Flow cytometry analysis of cells stably expressing PTHR1-GFP. HEK293 cell lines in suspension were incubated on ice with 100 nM VHH sortagged with Alexafluor647, pelleted by centrifugation, washed and analyzed. The VHH$_{GFP}$ used in this study binds both GFP and YFP (ref. 22, main text), at 100 nM it only weakly stained HEK293 cells stably expressing PTHR1$_{GFP}$. This weak staining is likely related to the inability of VHHGFP to tightly bind the pH-sensitive GFP variant known as pHluorin2 engrafted into the receptor(ref. 19, main text). c) HEK293 cells stably expressing PTHR1-GFP were treated with varied doses of the indicated peptides or conjugates and activation was assessed by measuring luminescence from a cAMP-activated luciferase variant. Values listed represent EC_{50} values (mean ± SD). Each value comes from ≥ 3 independent experiments. Further details, including the number of replicates for each measurement and the normalized maximal responses induced, are reported in Supplementary Table 1. “ND” indicates that the measurement was not made. “Inactive” indicates that the luminescence response measured at that concentration was less than 5% of the maximal response induced for that cell line.

Peptide	$[M+H]_{calc}$	$[M+H]_{obs}$
PTH(1-9)	1127.6	1127.1
PTH(1-10)	1255.6	1255.1
PTH(1-11)	1411.7	1411.2
PTH(1-14)	1796.9	1796.3
PTH(1-34)	4218.2	4217.5
PTH(1-9)-dbco	1554.8	1554.2
PTH(1-10)-dbco	1682.8	1682.2
PTH(1-11)-dbco	1838.9	1839
PTH(1-14)-dbco	2224.1	2223.5
PTH(1-34)-dbco	4645.4	4644.6
PTH(1-11)-PEG3-dbco	2042	2041.4
PTH(1-34)-PEG3-dbco	4848.5	4847.7
G_3-PTH(1-14)	1865	1864.2

Supplementary Figure 2. Confirmation of peptide identity using mass spectrometry. Peptides were analyzed by LC/MS as described in methods. Calculated masses ($[M+H]_{calc}$) refers to the monoisotopic mass of a singly protonated species. The masses recorded using mass spectrometry are labeled as $[M+H]_{obs}$.

Conjugate	MW_{calc}	MW_{obs}
VHH_{GFP}-G3-Lys(biotin)AhxLys(azide)	n/a	14080
VHH_{GFP}-PTH(1-9)	15635	15635
VHH_{GFP}-PTH(1-10)	15763	15765
VHH_{GFP}-PTH(1-11)	15919	15920
VHH_{GFP}-PTH(1-14)	16304	16305
VHH_{GFP}-G3-PTH(1-14)	15135	15135
VHH_{GFP}-PTH(1-34)-PEG	18929	18930
VHH_{6E}-G3-Lys(biotin)AhxLys(azide)	n/a	13325
VHH_{6E}-PTH(1-9)	14880	14895
VHH_{6E}-PTH(1-10)	15008	15010
VHH_{6E}-PTH(1-11)	15164	15165
VHH_{6E}-PTH(1-11)-PEG	15367	15375
VHH_{6E}-PTH(1-14)	15549	15550
VHH_{6E}-PTH(1-34)-PEG	18174	18175
VHH_{6E}-G3-PTH(1-14)	14380	14380
VHH_{PTHR}-PTH(1-9)	16505	16505
VHH_{PTHR}-PTH(1-10)	16633	16635
VHH_{PTHR}-PTH(1-11)	16789	16790
VHH_{PTHR}-PTH(1-14)	17174	17175
VHH_{PTHR}-G3-Lys(biotin)AhxLys(azide)	n/a	14950
VHH_{PTHR}-G3-PTH(1-14)	16005	16005

Supplementary Figure 3. Confirmation of VHH-peptide conjugate identity using mass spectrometry. VHH-peptide conjugates were analyzed by LC/MS as described in methods. Deconvolution calculations were used to provide the observed values. MW_{calc} refers to the calculated average molecular weight and MW_{obs} refers to the molecular weight recorded by mass spectrometry.

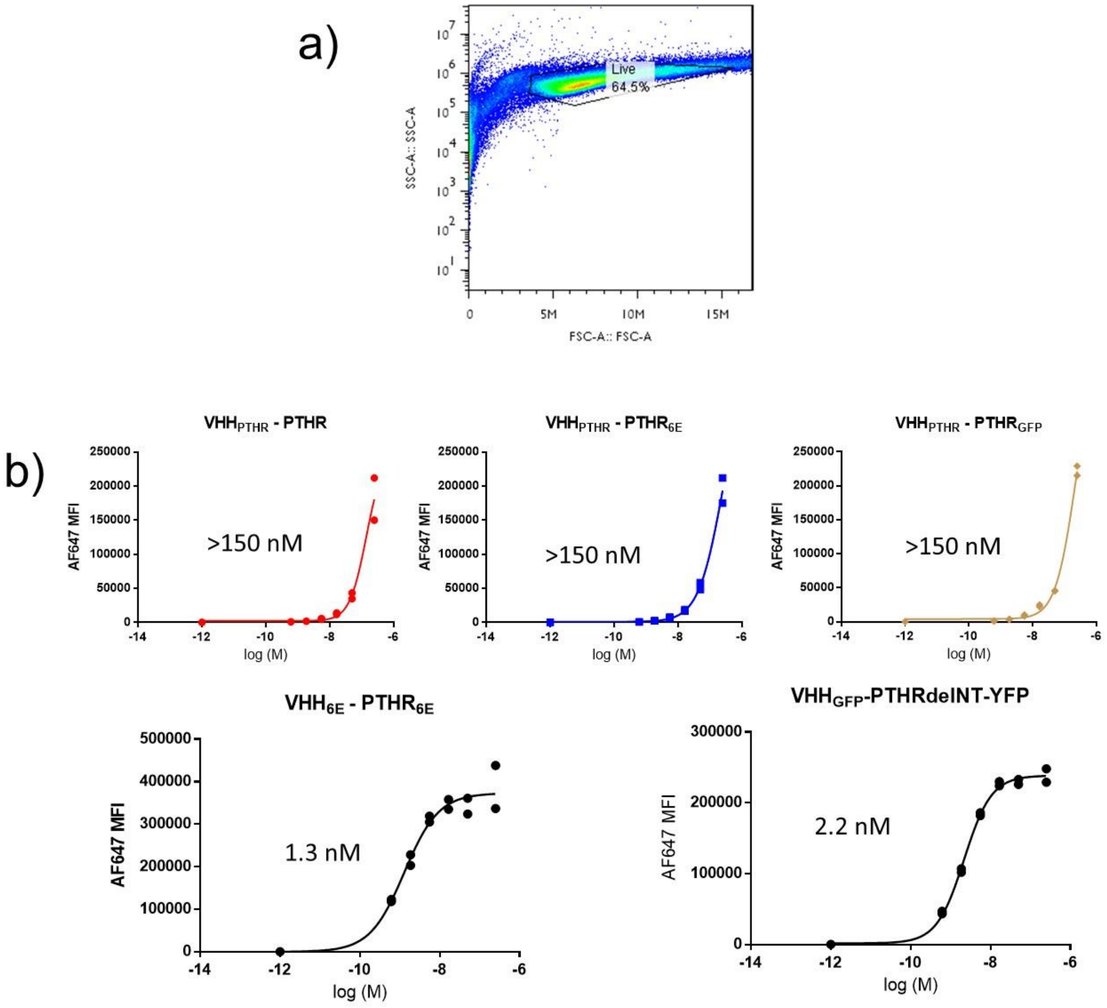

Supplementary Figure 4. Assessment of VHH binding to PTHR1 variants by flow cytometry. Cells dislodged from tissue culture plates using trypsinization were incubated with varied concentrations of VHHs sortagged with AlexaFluor647 on ice for 1 h. Cells were centrifuged, washed, and analyzed by flow cytometry via gating of intact cells (live gate) based on forward scatter/side scatter profiles (panel **a**). (**b**) Data points represent median fluorescent intensity values (individual data points shown, n = 2 independent replicates per condition). Connecting curves are the result of fitting a sigmoidal dose-response model to the data points. Precise estimates for VHH_{PTHR} binding cannot be provided since the curve does not plateau at the highest concentration tested.

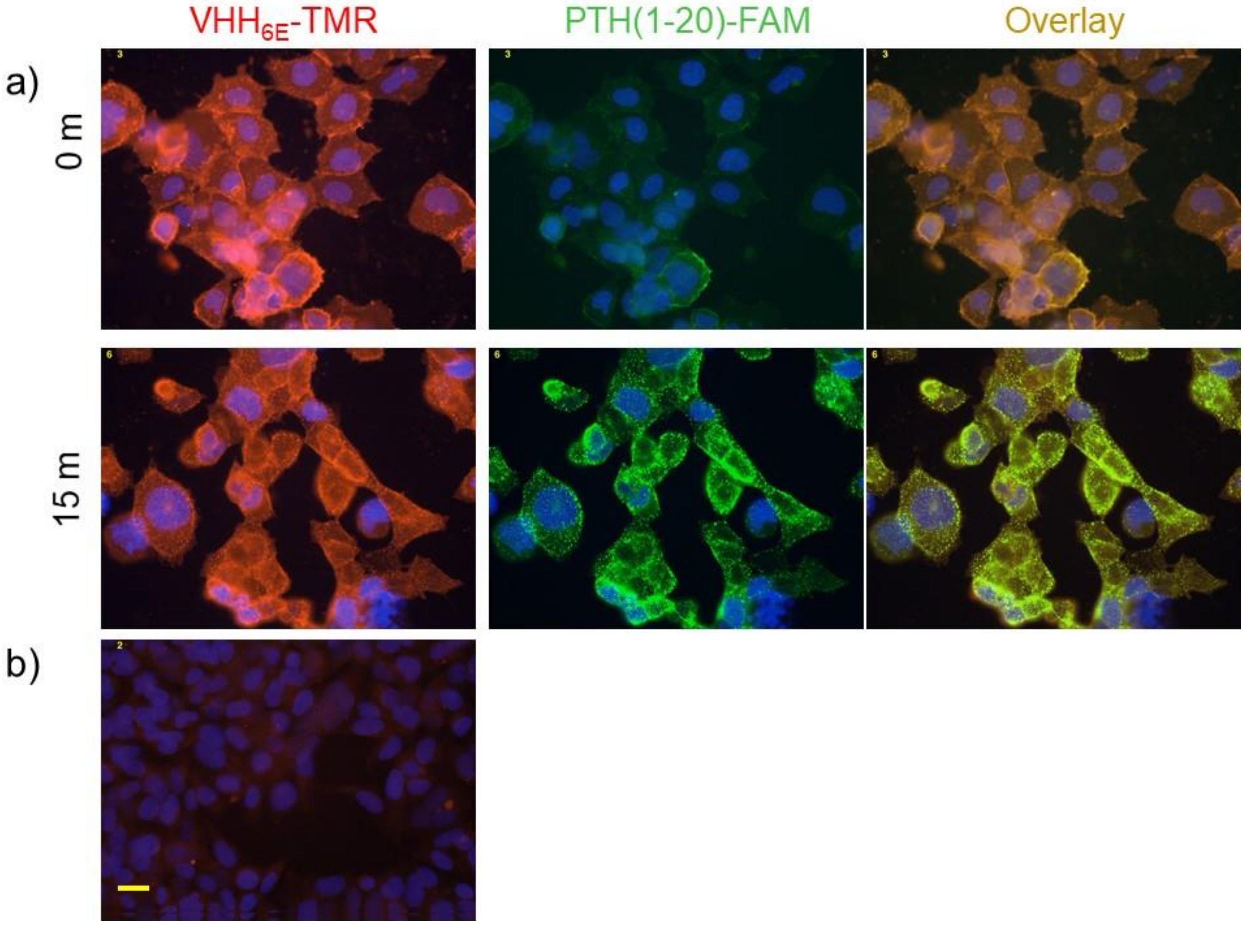

Supplementary Figure 5: Assessment of VHH_{6E} and PTH(1-20) binding to $PTHR1_{6E}$using microscopy. a) Adherent HEK293 cells expressing human PTHR1 were stained on ice with 300 nM VHH_{6E}-TMR and 30 nM PTH(1-20)-FAM for 30 minutes. Following staining cells were washed and treated with fixative in preparation for image acquisition either immediately after staining (0 m) or following a 15 minute incubation in medium at room temperature (15 m). Nuclei were visualized using DAPI staining. b) Adherent HEK293 cells not expressing PTHR1 were stained with VHH_{6E}-TMR and VHH_{PTHR}-TMR (300 nM each) and imaged as in panel a. The scale bar (yellow, 20 µm) is found in the bottom left corner of panel b and is applicable to all images. This experiment was repeated twice with similar results.

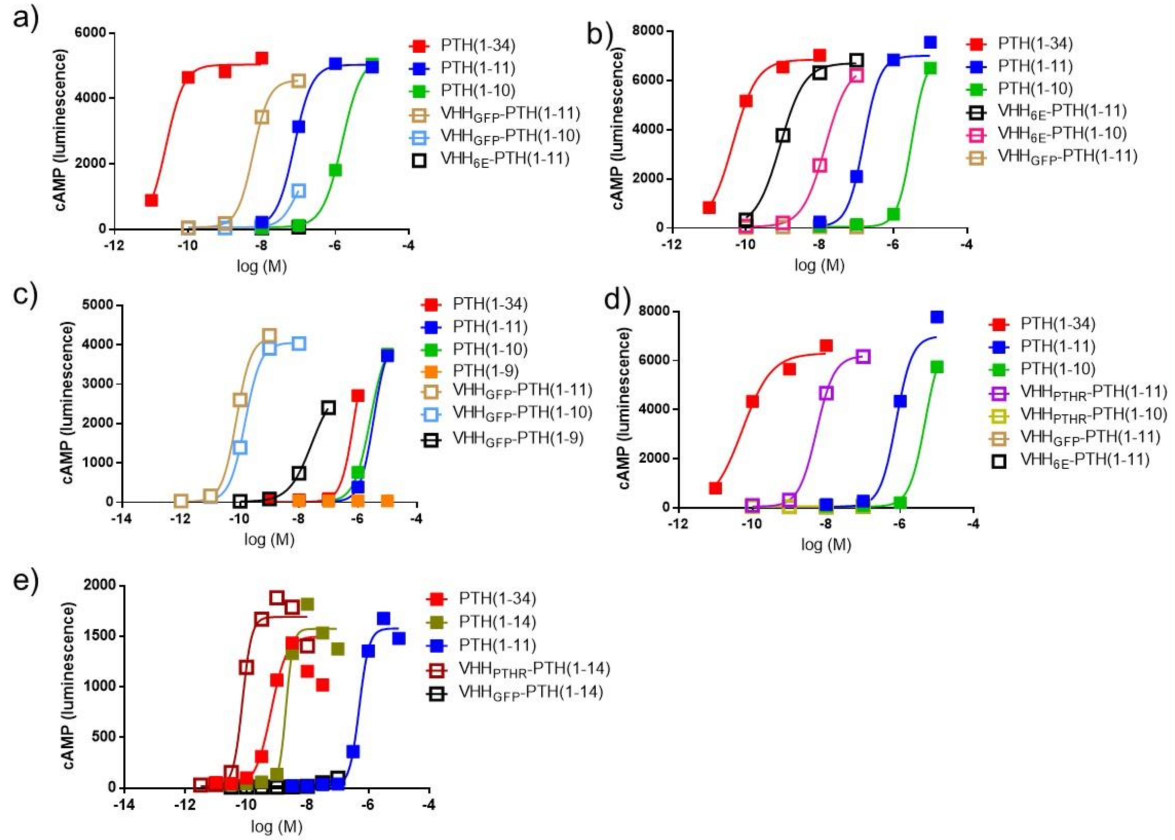

Supplementary Figure 6. Representative dose-response curves for cAMP induction in HEK293 cell lines. Varying concentrations of ligands were added to clonal HEK293-derived cell lines stably expressing the indicated receptor and the time course of luminescence response was recorded using a plate reader. The maximal luminescence response (observed 12–16 min after ligand addition) was used to construct dose-response data sets . Data points represent mean from two technical replicates and connecting lines result from the fit of a four-parameter sigmoidal dose-response model. Cell lines stably express (a) PTHR1$_{GFP}$, (b) PTHR1$_{6E}$, (c) PTHR1$_{YFP\Delta ECD}$, or (d-e) human PTHR1. Complete descriptions of data including all replicates are found in Supplementary Table 1.

a)

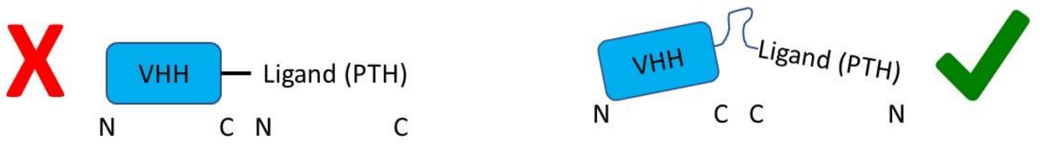

b)

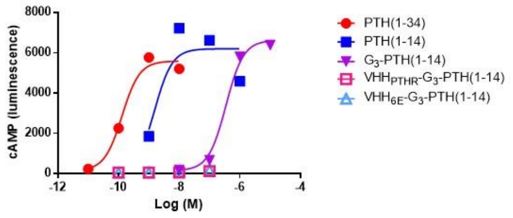

c)

Peptide/conjugate	EC_{50}, nM (± SD)			n
PTH(1-34)	0.38	±	0.23	4
PTH(1-14)	2.8	±	1.5	4
G_3-PTH(1-14)	208	±	135	4
VHH_{PTHR}-G_3-PTH(1-14)	No activity at 100 nM			3
VHH_{6E}-G_3-PTH(1-14)	No activity at 100 nM			3

Supplementary Figure 7. Modification of the N-terminus of PTH degrades activity. (**a**) Schematic comparison of the topology of the two types of conjugates tested. (**b**) Representative dose-response curves for activation of human PTHR1 by indicated peptides or conjugates run as described in methods. Data points indicate mean from two technical replicates and connecting lines result from the fit of a four-parameter sigmoidal dose-response model. (**c**) Tabulation of cAMP induction potencies. Note that these data correspond to a subset of the complete data set described Supplementary Table 1.

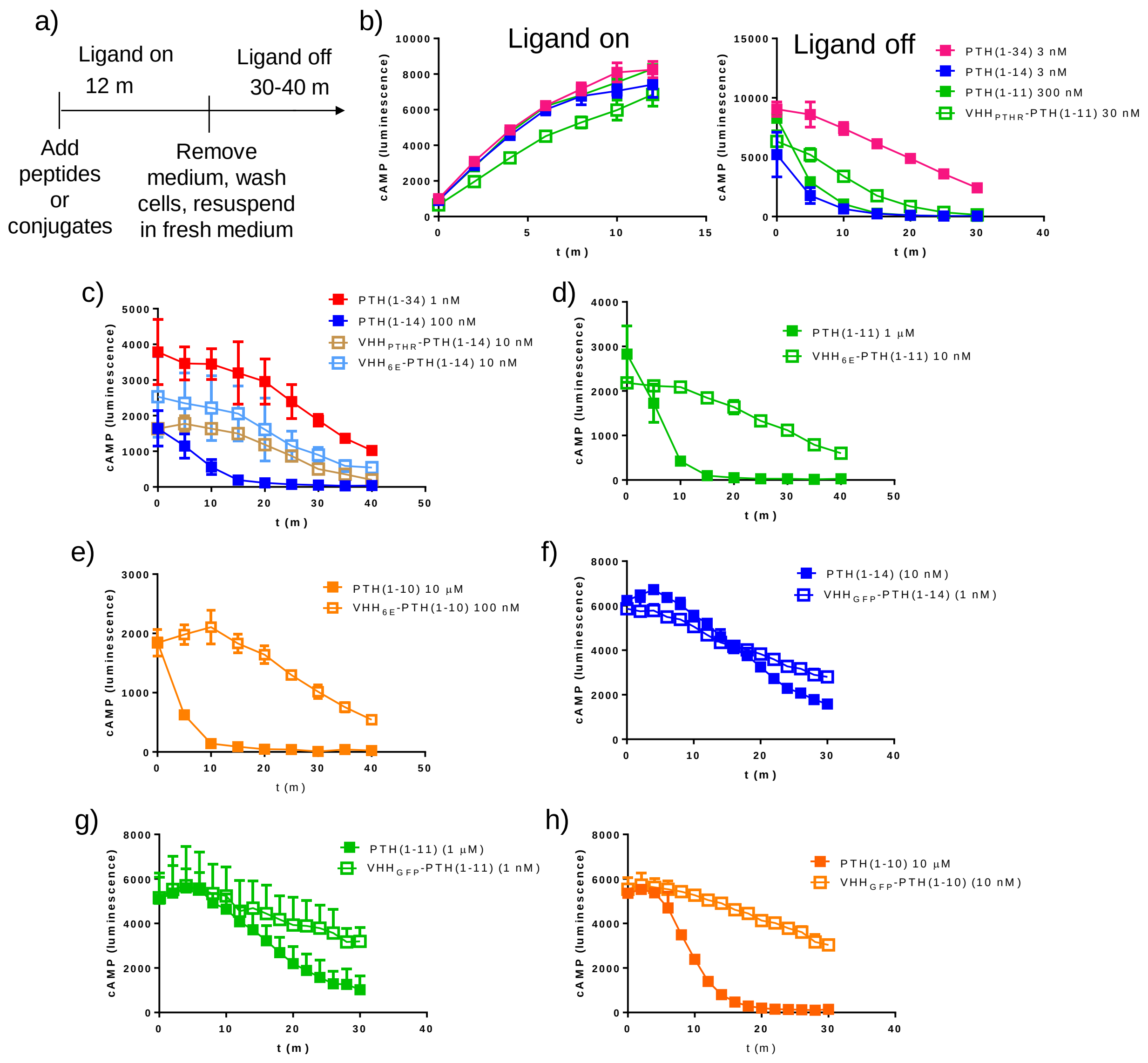

Supplementary Figure 8. VHH anchoring of PTH fragments prolongs cAMP signaling. Cells were treated with peptides or conjugates at concentrations listed in the legend for each figure to stimulate cAMP responses as described in methods. (**a**) Scheme describing workflow for cAMP kinetics experiments. cAMP responses were recorded every two minutes following addition of peptide and after washout of free peptide. The time needed for medium removal, washing of cells, and resuspension in fresh medium spans approximately 2 minutes. (**b**) Representative plots of the kinetics of cAMP-induced signal production (left) and signal cessation after removal of medium containing ligand from (right) hPTHR1 expressing HEK293 cells. Ligands were used at the minimal concentration that stimulated near maximal cAMP responses to minimize effects from non-specific adherence. Lines connect data points and only serve to guide the eye. The ligand on phase was omitted from panels c-h but each ligand tested induced a similar cAMP response prior to washout (data not shown). (**c-h**) Ligand off phase following stimulation of cells expressing (**c-e**) $PTHR1_{6E}$ or (**f-h**) $PTHR1_{YFP\Delta ECD}$. Data from individual cell lines are separated into separate panels for clarity. Data points indicate mean ± SD from three independent replicates.

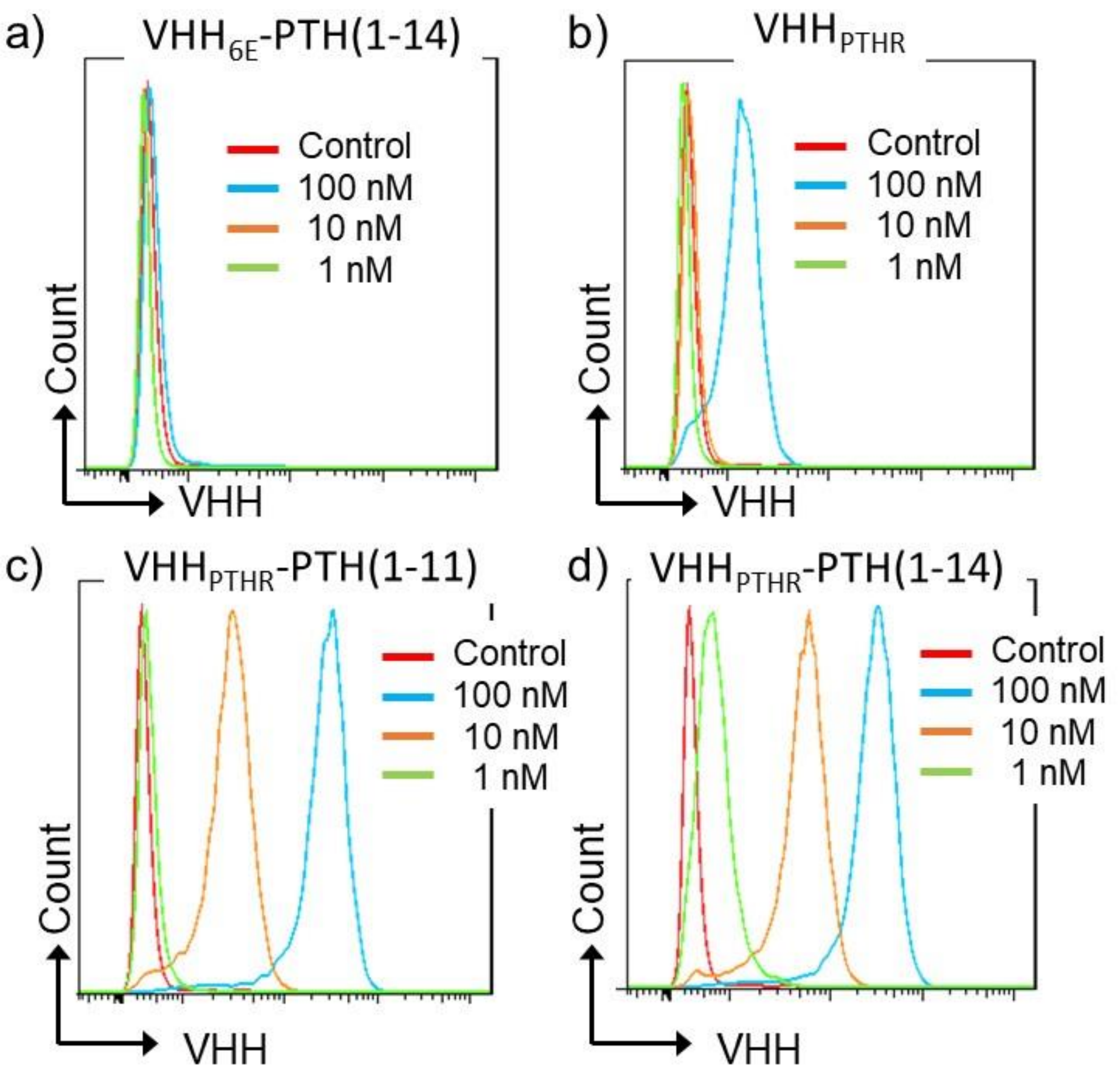

Supplementary Figure 9. Variation in binding caused by peptide conjugation. HEK293 cells expressing PTHR1 were stained with conjugates indicated in panels a-d at concentrations listed in legends and prepared for analysis by flow cytometry as described in methods. The control staining condition for each panel was staining with VHH_{6E}-biotin-azide used at a concentration of 100 nM. In panel b, VHH_{PTHR}-biotin-azide was used for staining.

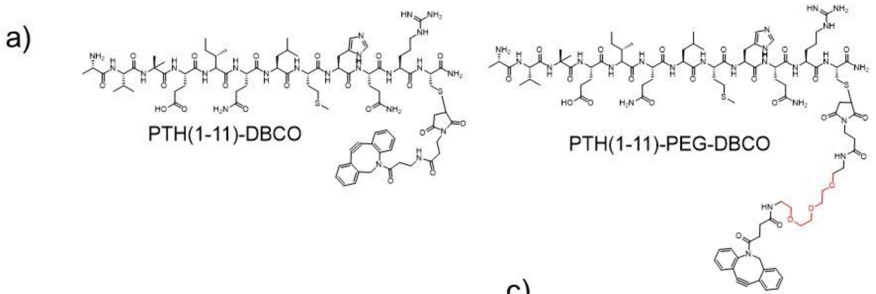

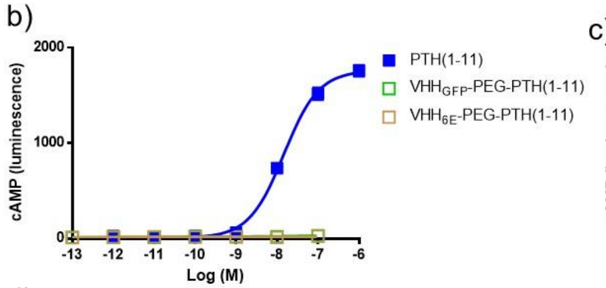

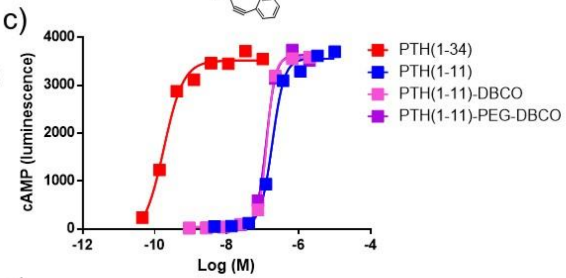

d)

Peptide/conjugate	EC_{50}, nM (± SD)			n
PTH(1-34)	0.67	±	0.64	6
PTH(1-11)	336	±	208	7
PTH(1-11)-DBCO	65	±	48	4
PTH(1-11)-PEG-DBCO	145	±	17	3
VHH_{GFP}-PEG-PTH(1-11)	No activity at 100 nM			3
VHH_{6E}-PEG-PTH(1-11)	No activity at 100 nM			2

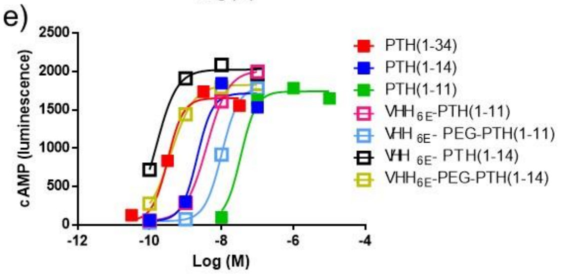

f)

Peptide/conjugate	EC_{50}, nM (± SD)			n
PTH(1-34)	1.3	±	0.98	3
PTH(1-14)	3.4	±	2	3
PTH(1-11)	94	±	74	3
VHH_{6E}-PTH(1-14)	0.43	±	0.24	3
VHH_{6E}-PEG-PTH(1-14)	0.81	±	0.36	3
VHH_{6E}-PTH(1-11)	6.9	±	2.6	3
VHH_{6E}-PEG-PTH(1-11)	21	±	9.8	3

Supplementary Figure 10. Impact of DBCO conjugation and PEG linker insertion on bioactivity. Peptides and VHH-peptide conjugates were assessed for cAMP induction in HEK293 cell lines as described in methods. Data points represent mean from two technical replicates in a single experiment and connecting lines result from the fit of a four-parameter sigmoidal dose-response model. (**a**) Structure of PTH(1-11) fused to either DBCO or PEG_3-DBCO to illustrate connectivity. Atoms corresponding to the PEG linker are highlighted in red. (**b-c**) Representative dose-response curves for stimulation of human PTHR1. (**b**) Insertion of a PEG_3linker does not enable activation of receptors not bound by VHHs by VHH-PTH(1-11) conjugates. (**c**) Attachment of DBCO or PEG-DBCO to PTH(1-11) does not substantially alter receptor activation properties. (**d**) Tabulation of experimental results for activation of hPTHR1 by PTH and conjugates. These data are distinct from those presented in Table 1. (**e**) Representative dose-response curve for stimulation of $PTHR1_{6E}$ by DBCO and PEG-DBCO conjugates of PTH fragments. Insertion of a PEG_3linker does not substantially alter receptor activation properties. (**f**) Tabulation of experimental results for activation of PTHR1-6E by PTH and conjugates. These data are distinct from those presented in Table 1.

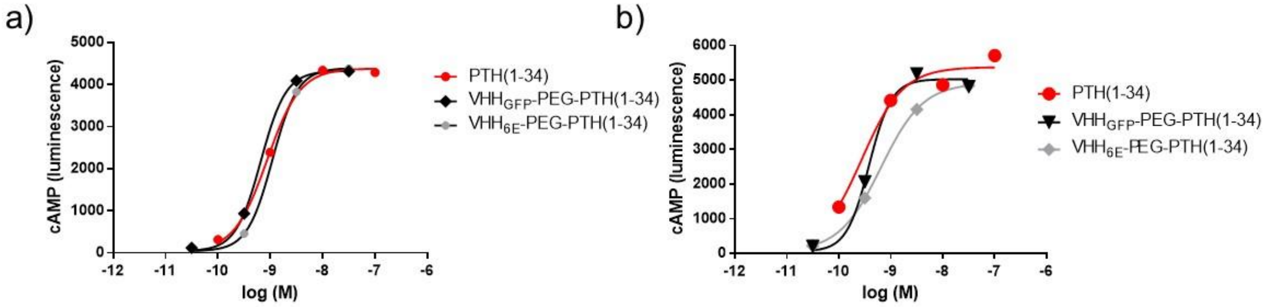

Peptide/conjugate	EC_{50}, nM (± SD)			n
PTH(1-34)	0.56	±	0.29	3
VHH_{GFP}-PEG-PTH(1-34)	0.52	±	0.12	3
VHH_{6E}-PEG-PTH(1-34)	0.83	±	0.29	3

Peptide/conjugate	EC_{50}, nM (± SD)			n
PTH(1-34)	1.05	±	1.26	4
VHH_{GFP}-PEG-PTH(1-34	0.90	±	0.78	3
VHH_{6E}-PEG-PTH(1-34)	1.44	±	1.14	4

Supplementary Figure 11. VHH conjugation does not affect signaling capacity of PTH(1-34). PTH(1-34)-Cys was conjugated to VHH with an intervening PEG_3 linker as described in Figure 2 and Supplementary Figure 10. The induction of cAMP responses was assessed in cell lines expressing (**a**) hPTHR1 or (**b**) $PTHR1_{6E}$. Representative dose-response curves are shown in which data points indicate mean from two technical replicates in a single experiment and connecting lines result from the fit of a four-parameter sigmoidal dose-response model. Composite results are tabulated below the dose-response curves.

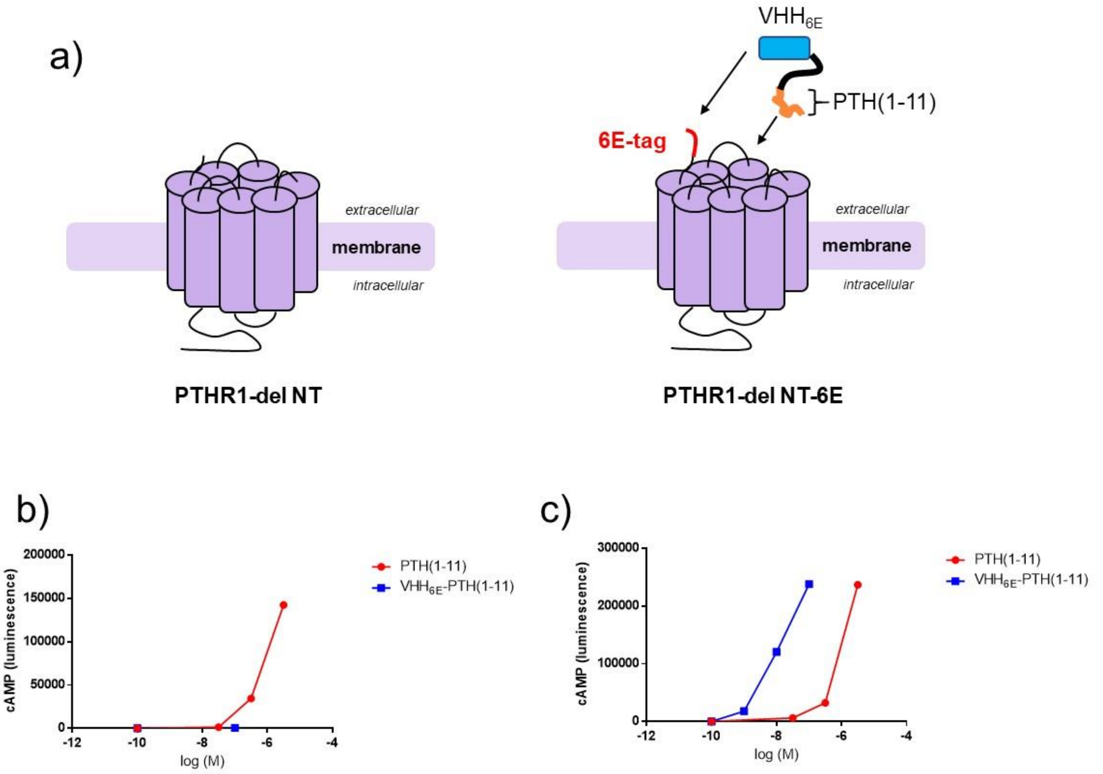

Supplementary Figure 12. Targeting PTHR1 lacking extracellular domain. HEK293 cells stably expressing cAMP-responsive luciferase were transiently transfected with either rat PTHR1 lacking extracellular domain (PTHR1-delNT) or a construct with the 6E tag inserted in place of the extracellular domain (PTHR1-delNT-6E). See Supplementary Figure 19 for sequences. (**a**) Schematic of receptor constructs and targeting strategy. (**b-c**) Characterization of cAMP responses induced by PTH(1-11) or VHH_{6E}-PTH(1-11) on HEK293 cells transected with (**b**) PTHR1-delNT or (**c**) PTHR1-delNT-6E. Data points represent mean duplicate wells. Each experiment was verified in three independent experiments (data not shown). Lines on the graph are not from the fitting of a model and only serve to guide the eye. PTH(1-11)-Cys is the same sequence as listed in Figure 2. cAMP response assays were performed as described in methods.

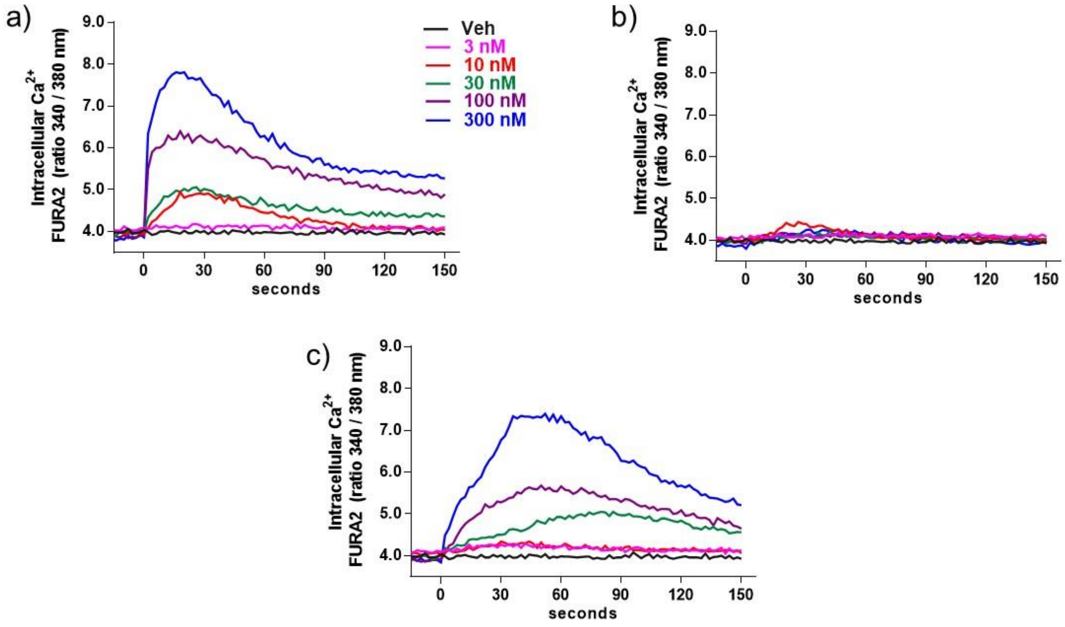

Supplementary Figure 13. Measurement of cytoplasmic calcium mobilization by PTHR1 agonists. HEK293/PTHR1 cells were loaded with FURA2-AM, then stimulated with (**a**) PTH(1-34), (**b**) VHH$_{PTHR}$-PTH(1-11) or (**c**) VHH$_{PTHR}$-PTH(1-14) at time zero as described in the methods section. The colors used to represent each concentration are held consistent in each panel. Data points indicate mean from two independent measurements.

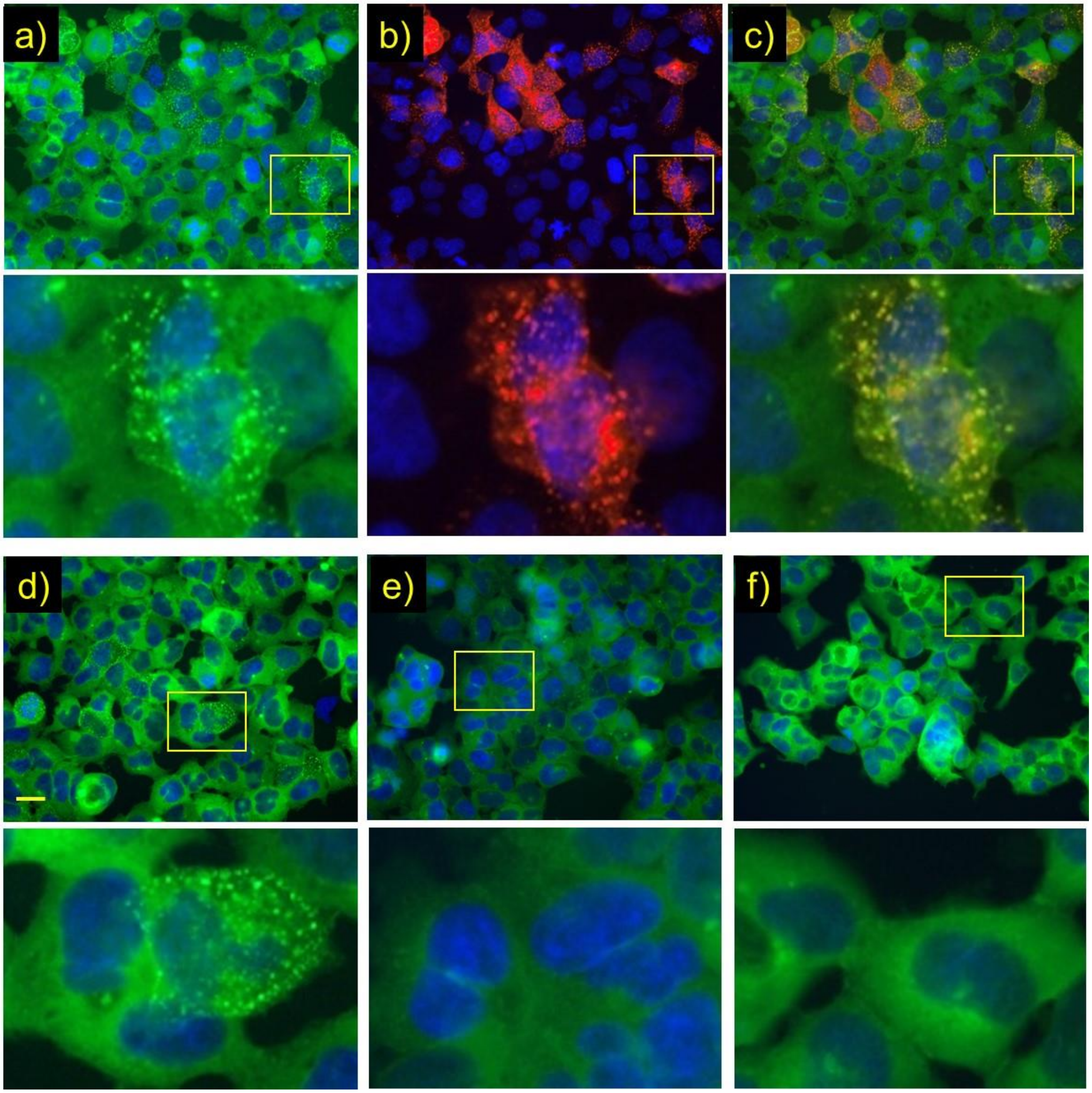

Supplementary Figure 14. Assessment of β-arrestin recruitment. A HEK293-derived cell line stably expressing a β-arrestin2-YFP fusion[1] (green) was transiently transfected with human PTHR1. Some panels show cells stained with PTH(1-34)-tetramethylrhoadmine conjugate [PTH(1-34)-TMR] (red) and the nuclei of all cells were counterstained with DAPI (blue). Insets in rectangles are magnified below the main image. The ligands or VHHs (and concentrations) applied to these cells are (**a-c**) PTH(1-34)-TMR (30 nM), (**d**) VHH_{PTHR}-PTH(1-14) (100 nM), (**e**) VHH_{PTHR} (100 nM), and (**f**) Vehicle. Panels a-c show the same field of view with signal for (**a**) β-arrestin2-YFP, (**b**) PTH(1-34)-TMR, and (**c**) overlay. Note that only a portion of the cells appear to be transfected with PTHR1 as indicated by PTH(1-34)-TMR staining (panel b). Signals for β-arrestin2-YFP and PTH(1-34)-TMR colocalize in puncta (panel c, inset Punctate β-arrestin2-YFP signals are not observed in untransfected (PTH(1-34)-TMR negative) cells of panel c (inset, left) or in any of the cells of panels e and f treated with VHH_{PTHR} or vehicle, respectively. Transfected cells

were incubated with indicated ligands or VHHs at room temperature for 30 minutes. This solution was aspirated, and the cells were washed twice, fixed with paraformaldehyde, and imaged as described in methods. For each panel the bottom image corresponds to an expanded version of the inset marked by the rectangle in the top image. The scale bar (20 µm) is found in the bottom left corner of panel d and is applicable to panels a-c (but not expanded insets). This experiment was repeated twice with similar results.

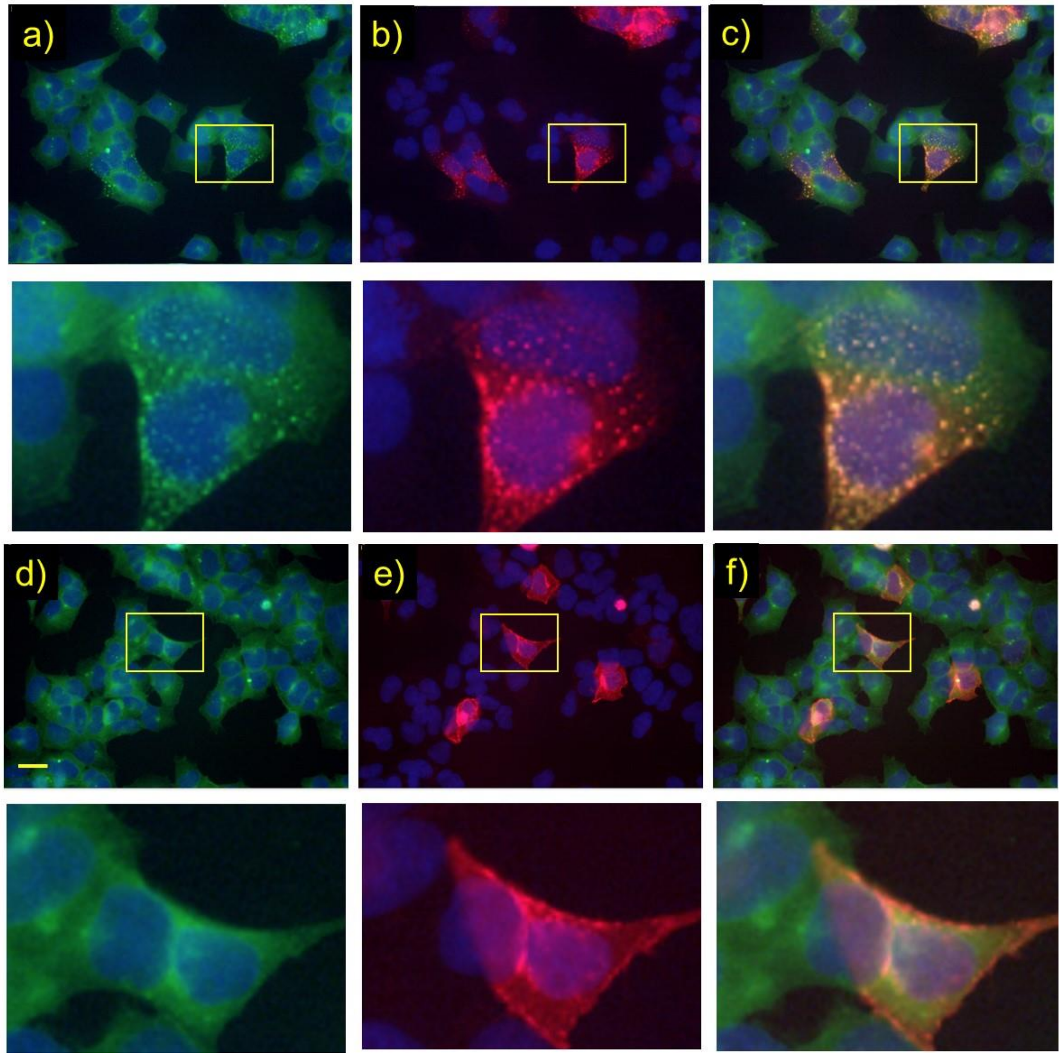

Supplementary Figure 15. Assessment of β-arrestin recruitment stimulated by VHH$_{PTHR}$-PTH(1-14). A HEK293-derived cell line stably expressing a β-arrestin2-YFP fusion[1] (green) was transiently transfected with human PTHR1 with an HA epitope tag incorporated into exon 2 (Supplementary Figure 19). Insets in rectangles are magnified below the main image. Some panels show cells stained with fluorophore-conjugated (Alexafluor594) anti-HA antibody (red) and the nuclei of all cells were counterstained with DAPI (blue). The ligands or VHHs (and concentrations) applied to these cells are (**a-c**) VHH$_{PTHR}$-PTH(1-14) (100 nM) or (**d-f**) VHH$_{PTHR}$ (100 nM). Panels a-c show the same field of view with signal for (**a**) β-arrestin2-YFP, (**b**) Anti-HA-AF594 (hPTHR1-HA), and (**c**) overlay. Panels d-f show the same field of view. Note that only a portion of the cells appear to be transfected with PTHR1 as indicated by anti-HA staining (panels b, e). Signals for β-arrestin2-YFP and PTHR1-HA colocalize in puncta in cells treated with

VHH$_{PTHR}$-PTH(1-14) (panel c) but not VHH$_{PTHR}$ (panel f). Transfected cells were incubated with indicated ligands or VHHs at room temperature for 30 minutes. This solution was aspirated, and the cells were washed twice, fixed with formalin, permeabilized (0.5% Triton X100), stained with anti-HA antibody, and imaged as described in methods. The scale bar (20 μm) is found in the bottom left corner of panel d and is applicable to panels a-c (but not expanded insets). This experiment was repeated twice with similar results.

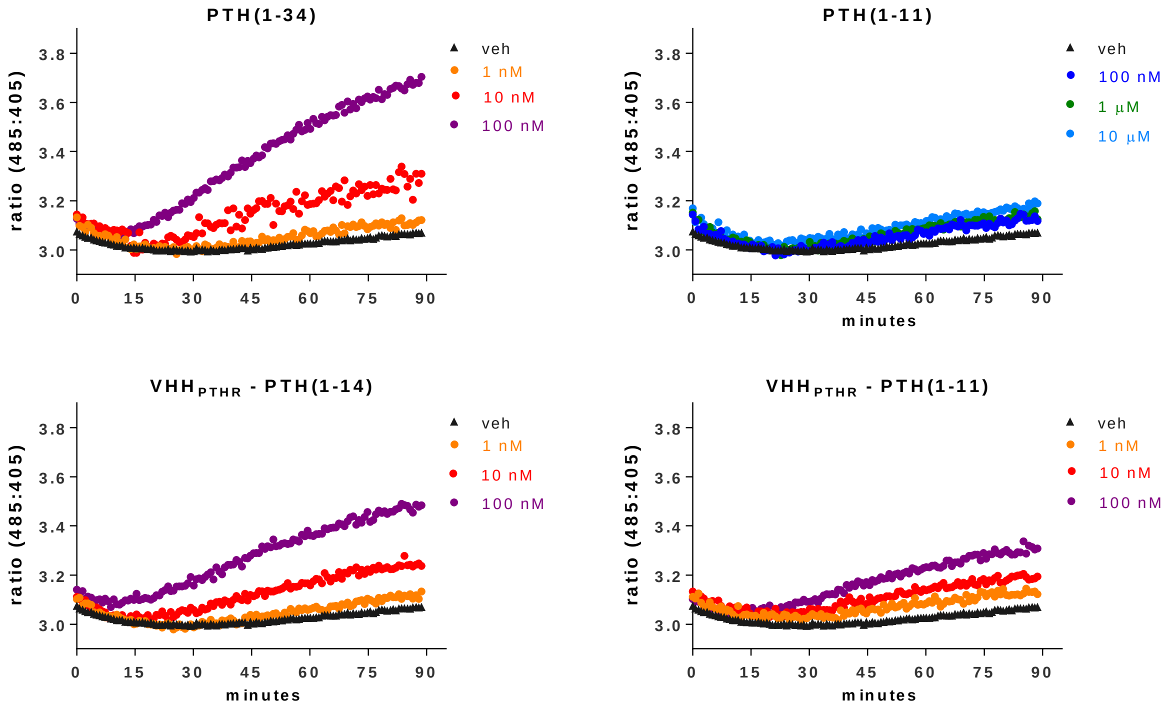

Supplementary Figure 16. Assessment PTHR1 internalization. A HEK293-dervied cell line stably expressing a PTHR1-GFP-pHluorin2 was treated with a PTH peptide or VHH-peptide doses at the indicated doses. The ratio of fluorescence intensity at 535 nm following excitation at either 485 nm or 405 nm was measured over time. Data points indicate mean from two technical replicates. These data are representative of two independent experiments.

a)

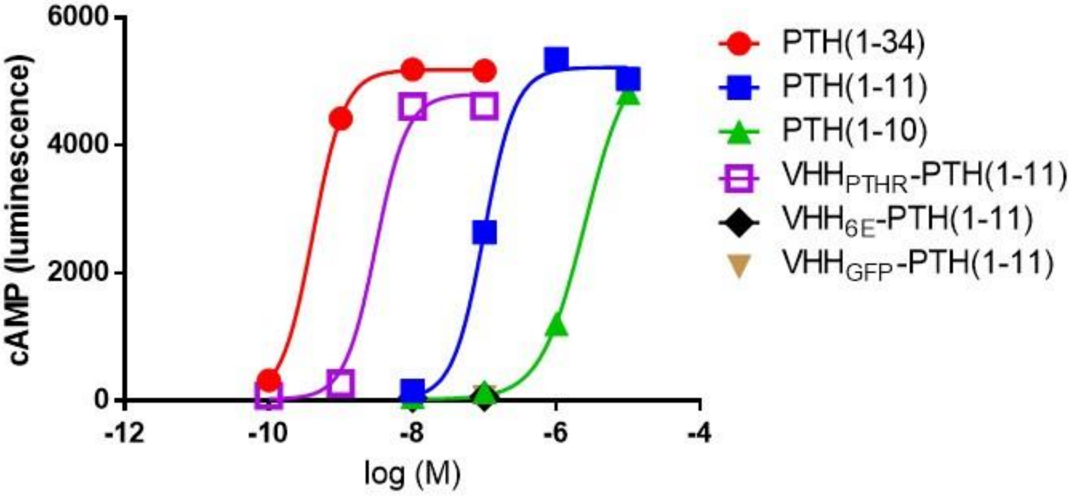

b)

**Peptide/conjugate**	**EC_{50} (nM ± SD)**			***n***
PTH(1-34)	0.28	±	0.12	3
PTH(1-11)	120.3	±	90.3	3
PTH(1-10)	2466	±	23	3
VHH$_{PTHR}$-PTH(1-11)	3.2	±	0.9	3
VHH$_{6E}$-PTH(1-11)	>100 nM			2
VHH$_{GFP}$-PTH(1-11)	>100 nM			2

Supplementary Figure 17. VHH$_{PTHR}$ conjugation potentiates PTH fragment activation of rat PTHR1. HEK293 cells stably expressing rat PTHR1 were stimulated with peptide or conjugate as described in methods. (**a**) Representative dose-response curve for a stimulation of rPTHR1 by peptides or VHH-PTH(1-11) conjugates in a single experiment. Data points indicate mean from two technical replicate measurements and connecting lines result from the fit of a four-parameter sigmoidal dose-response model. (**b**) Tabulation of composite results from rPTHR1 cAMP stimulation assays.

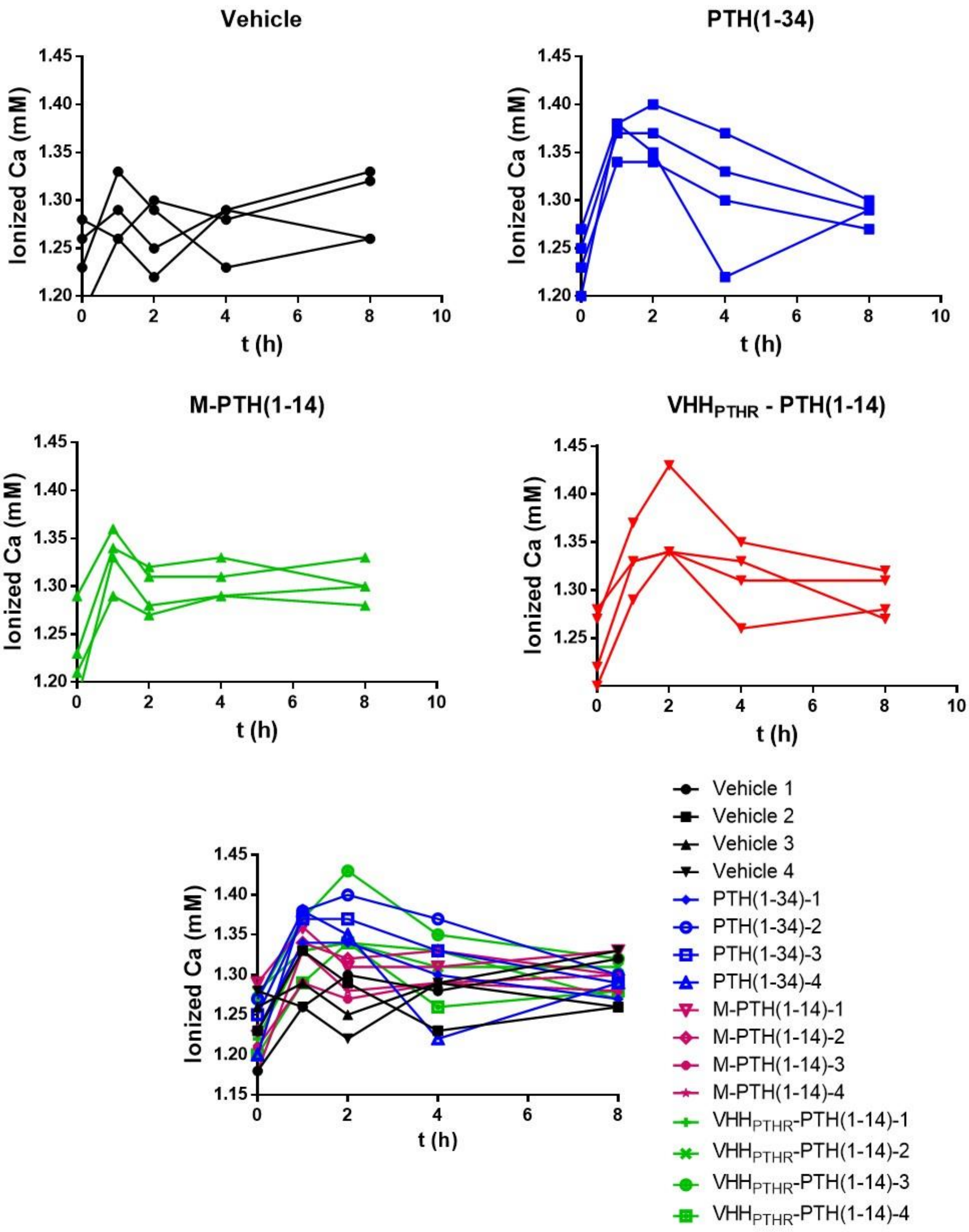

Supplementary Figure 18. Calcemic responses for individual mice. Each trace represents the calcium measurement recorded over time in a single mouse as described in Figure 5 in the main text.

```
hPTHR1                      MGTARIAPGL ALLLCCPVLS SAYALVDADD VMTKEEQIFL LHRAQAQCEK
hPTHR1-GFP                  MGTARIAPGL ALLLCCPVLS SAYALVDADD VMTKEEQIFL LHRAQAQCEK
rPTHR1-delNT26-181_         MGAARIAPSL ALLLCCPVLS SAYAL..... ~~~~~~~~~~ ~~~~~~~~~~
hPTHR1-delNT-6E             MGAARIAPSL ALLLCCPVLS SAY....QAD QEAKELARQI SGGGG~~~~~
hPTHR1_6E                   MGTARIAPGL ALLLCCPVLS SAYALVDADD VMTKEEQIFL LHRAQAQCEK
PTHR1-delNT-YFP             MGTARIAPGL ALLLCCPVLS SAYAL~~~~~ ~~~~~~~~~~ ~~~~~~~~~~

                            51                                                100
hPTHR1                      RLKEVLQRPA SIMESDKGWT SASTSGKPRK DKASGKLYPE SEED......
hPTHR1-GFP                  RLKEVLQRPA SIMESDKGWT SASTSGKPRK DKASGKLYPE SEEDKMSKGE
rPTHR1-delNT26-181_         ~~~~~~~~~~ ~~~~~~~~~~ ~~~~~~~~~~ ~~~~~~~~~~ ~~~~~~~~~~
hPTHR1-delNT-6E             ~~~~~~~~~~ ~~~~~~~~~~ ~~~~~~~~~~ ~~~~~~~~~~ ~~~~~~~~~~
hPTHR1_6E                   RLKEVLQRPA SIMESDKGWT QADQEAKELA RQISGKLYPE SEED~~~~~~
hPTHR1-delNT-YFP            ~~~~~~~~~~ ~~~~~~~~~~ ~~~~~~~~~~ ~~~~~~~~~~ ~GATMVSKGE

                            101                                               150
hPTHR1                      .......... .......... .......... .......... ..........
hPTHR1-GFP                  ELFTGVVPIL VELDGDVNGH KFSVSGEGEG DATYGKLTLK FICTTGKLPV
rPTHR1-delNT26-181_         ~~~~~~~~~~ ~~~~~~~~~~ ~~~~~~~~~~ ~~~~~~~~~~ ~~~~~~~~~~
hPTHR1-delNT-6E             ~~~~~~~~~~ ~~~~~~~~~~ ~~~~~~~~~~ ~~~~~~~~~~ ~~~~~~~~~~
hPTHR1_6E                   ~~~~~~~~~~ ~~~~~~~~~~ ~~~~~~~~~~ ~~~~~~~~~~ ~~~~~~~~~~
hPTHR1-delNT-YFP            ELFTGVVPIL VELDGDVNGH KFSVSGEGEG DATYGKLTLK LICTTGKLPV

                            151                                               200
hPTHR1                      .......... .......... .......... .......... ..........
hPTHR1-GFP                  PWPTLVTTLS YGVQCFSRYP DHMKQHDFFK SAMPEGYVQE RTIFFKDDGN
rPTHR1-delNT26-181_         ~~~~~~~~~~ ~~~~~~~~~~ ~~~~~~~~~~ ~~~~~~~~~~ ~~~~~~~~~~
rPTHR1-delNT-6E             ~~~~~~~~~~ ~~~~~~~~~~ ~~~~~~~~~~ ~~~~~~~~~~ ~~~~~~~~~~
hPTHR1_6E                   ~~~~~~~~~~ ~~~~~~~~~~ ~~~~~~~~~~ ~~~~~~~~~~ ~~~~~~~~~~
hPTHR1-delNT-YFP            PWPTLVTTLG YGVQCFARYP DHMKQHDFFK SAMPEGYVQE RTIFFKDDGN

                            201                                               250
hPTHR1                      .......... .......... .......... .......... ..........
hPTHR1-GFP                  YKTRAEVKFE GDTLVNRIEL KGIDFKEDGN ILGHKLEYNY NEHLVYIMAD
rPTHR1-delNT26-181_         ~~~~~~~~~~ ~~~~~~~~~~ ~~~~~~~~~~ ~~~~~~~~~~ ~~~~~~~~~~
hPTHR1-delNT-6E             ~~~~~~~~~~ ~~~~~~~~~~ ~~~~~~~~~~ ~~~~~~~~~~ ~~~~~~~~~~
hPTHR1_6E                   ~~~~~~~~~~ ~~~~~~~~~~ ~~~~~~~~~~ ~~~~~~~~~~ ~~~~~~~~~~
PTHR1-delNT-YFP             YKTRAEVKFE GDTLVNRIEL KGIDFKEDGN ILGHKLEYNY NSHNVYITAD

                            251                                               300
hPTHR1                      .......... .......... .......... .......... ..........
hPTHR1-GFP                  KQKNGTKAIF QVHHNIEDGS VQLADHYQQN TPIGDGPVLL PDNHYLHTQS
rPTHR1-delNT26-181_         ~~~~~~~~~~ ~~~~~~~~~~ ~~~~~~~~~~ ~~~~~~~~~~ ~~~~~~~~~~
rPTHR1-delNT-6E             ~~~~~~~~~~ ~~~~~~~~~~ ~~~~~~~~~~ ~~~~~~~~~~ ~~~~~~~~~~
hPTHR1_6E                   ~~~~~~~~~~ ~~~~~~~~~~ ~~~~~~~~~~ ~~~~~~~~~~ ~~~~~~~~~~
hPTHR1-delNT-YFP            KQKNGIKANF KIRHNIEDGG VQLADHYQQN TPIGDGPVLL PDNHYLSYQS

                            301                                               350
hPTHR1                      .......... .......... .......... ..KEAPTGSR YRGRPCLPEW
hPTHR1-GFP                  ALSKDPNEKR DHMVLLEFVT AAGITHGMDE LYKEAPTGSR YRGRPCLPEW
rPTHR1-delNT26-181_         ~~~~~~~~~~ ~~~~~~~~~~ ~~~~~~~~~~ ~~~~~~~~~~ ~~~~~~~~~~
rPTHR1-delNT-6E             ~~~~~~~~~~ ~~~~~~~~~~ ~~~~~~~~~~ ~~~~~~~~~~ ~~~~~~~~~~
hPTHR1_6E                   ~~~~~~~~~~ ~~~~~~~~~~ ~~~~~~~~~~ ~~KEAPTGSR YRGRPCLPEW
hPTHR1-delNT-YFP            KLSKDPNEKR DHMVLLEFVT AAGITLGMDE LYK~~~~~~~ ~~~~~~~~~~

                            351                                               400
hPTHR1                      DHILCWPLGA PGEVVAVPCP DYIYDFNHKG HAYRRCDRNG SWELVPGHNR
hPTHR1-GFP                  DHILCWPLGA PGEVVAVPCP DYIYDFNHKG HAYRRCDRNG SWELVPGHNR
rPTHR1-delNT26-181_         ~~~~~~~~~~ ~~~~~~~~~~ ~~~~~~~~~~ .......... ..........
hPTHR1-delNT-6E             ~~~~~~~~~~ ~~~~~~~~~~ ~~~~~~~~~~ .......... ..........
hPTHR1_6E                   DHILCWPLGA PGEVVAVPCP DYIYDFNHKG HAYRRCDRNG SWELVPGHNR
hPTHR1-delNT-YFP            ~~~~~~~~~~ ~~~~~~~~~~ ~~~~~~~~~~ .......... ..........

                            401                                               450
hPTHR1                      TWANYSECVK FLTNETRERE VFDRLGMIYT VGYSVSLASL TVAVLILAYF
hPTHR1-GFP                  TWANYSECVK FLTNETRERE VFDRLGMIYT VGYSVSLASL TVAVLILAYF
rPTHR1-delNT26-181_         ~~~~~~~~~~ .........E VFDRLGMIYT VGYSMSLASL TVAVLILAYF
hPTHR1-delNT-6E             ~~~~~~~~~~ .........E VFDRLGMIYT VGYSMSLASL TVAVLILAYF
hPTHR1_6E                   TWANYSECVK FLTNETRERE VFDRLGMIYT VGYSVSLASL TVAVLILAYF
hPTHR1-delNT-YFP            ~~~~~~~~~~ ......GSGE VFDRLGMIYT VGYSMSLASL TVAVLILAYF

                            451                                               500
hPTHR1                      RRLHCTRNYI HMHLFLSFML RAVSIFVKDA VLYSGATLDE AERLTEEELR
hPTHR1-GFP                  RRLHCTRNYI HMHLFLSFML RAVSIFVKDA VLYSGATLDE AERLTEEELR
rPTHR1-delNT26-181_         RRLHCTRNYI HMHMFLSFML RAASIFVKDA VLYSGFTLDE AERLTEEELH
rPTHR1-delNT-HA             RRLHCTRNYI HMHLFLSFML RAVSIFVKDA VLYSGATLDE AERLTEEELR
hPTHR1_delNT-6E             RRLHCTRNYI HMHLFLSFML RAVSIFVKDA VLYSGATLDE AERLTEEELR
hPTHR1_6E                   RRLHCTRNYI HMHLFLSFML RAVSIFVKDA VLYSGATLDE AERLTEEELR
hPTHR1_delNT-YFP            RRLHCTRNYI HMHLFLSFML RAVSIFVKDA VLYSGATLDE AERLTEEELR

                            501                                               550
hPTHR1                      AIAQAPPPPA TAAAGYAGCR VAVTFFLYFL ATNYYWILVE GLYLHSLIFM
hPTHR1-GFP                  AIAQAPPPPA TAAAGYAGCR VAVTFFLYFL ATNYYWILVE GLYLHSLIFM
rPTHR1-delNT26-181_         IIAQVPPPPA AAAVGYAGCR VAVTFFLYFL ATNYYWILVE GLYLHSLIFM
hPTHR1-delNT-6E             AIAQAPPPPA TAAAGYAGCR VAVTFFLYFL ATNYYWILVE GLYLHSLIFM
hPTHR1_6E                   AIAQAPPPPA TAAAGYAGCR VAVTFFLYFL ATNYYWILVE GLYLHSLIFM
hPTHR1_delNT-YFP            AIAQAPPPPA TAAAGYAGCR VAVTFFLYFL ATNYYWILVE GLYLHSLIFM
```

```
                                 551                                                    600
hPTHR1                           AFFSEKKYLW GFTVFGWGLP AVFVAVWVSV RATLANTGCW DLSSGNKKWI
hPTHR1-GFP                       AFFSEKKYLW GFTVFGWGLP AVFVAVWVSV RATLANTGCW DLSSGNKKWI
rPTHR1-delNT26-181_              AFFSEKKYLW GFTIFGWGLP AVFVAVWVGV RATLANTGCW DLSSGHKKWI
hPTHR1-delNT-6E                  AFFSEKKYLW GFTVFGWGLP AVFVAVWVSV RATLANTGCW DLSSGNKKWI
hPTHR1_6E                        AFFSEKKYLW GFTVFGWGLP AVFVAVWVSV RATLANTGCW DLSSGNKKWI
hPTHR1_delNT-YFP                 AFFSEKKYLW GFTVFGWGLP AVFVAVWVSV RATLANTGCW DLSSGNKKWI


                                 601                                                    650
hPTHR1                           IQVPILASIV LNFILFINIV RVLATKLRET NAGRCDTRQQ YRKLLKSTLV
hPTHR1-GFP                       IQVPILASIV LNFILFINIV RVLATKLRET NAGRCDTRQQ YRKLLKSTLV
rPTHR1-delNT26-181_              IQVPILASVV LNFILFINII RVLATKLRET NAGRCDTRQQ YRKLLRSTLV
hPTHR1-delNT-6E                  IQVPILASIV LNFILFINIV RVLATKLRET NAGRCDTRQQ YRKLLKSTLV
hPTHR1_6E                        IQVPILASIV LNFILFINIV RVLATKLRET NAGRCDTRQQ YRKLLKSTLV
hPTHR1_delNT-YFP                 IQVPILASIV LNFILFINIV RVLATKLRET NAGRCDTRQQ YRKLLKSTLV


                                 651                                                    700
hPTHR1                           LMPLFGVHYI VFMATPYTEV SGTLWQVQMH YEMLFNSFQG FFVAIIYCFC
hPTHR1-GFP                       LMPLFGVHYI VFMATPYTEV SGTLWQVQMH YEMLFNSFQG FFVAIIYCFC
rPTHR1-delNT26-181_              LVPLFGVHYT VFMALPYTEV SGTLWQIQMH YEMLFNSFQG FFVAIIYCFC
hPTHR1-delNT-6E                  LMPLFGVHYI VFMATPYTEV SGTLWQVQMH YEMLFNSFQG FFVAIIYCFC
hPTHR1_6E                        LMPLFGVHYI VFMATPYTEV SGTLWQVQMH YEMLFNSFQG FFVAIIYCFC
hPTHR1_delNT-YFP                 LMPLFGVHYI VFMATPYTEV SGTLWQVQMH YEMLFNSFQG FFVAIIYCFC


                                 701                                                    750
hPTHR1                           NGEVQAEIKK SWSRWTLALD FKRKARSGSS SYSYGPMVSH TSVTNVGPRV
hPTHR1-GFP                       NGEVQAEIKK SWSRWTLALD FKRKARSGSS SYSYGPMVSH TSVTNVGPRV
rPTHR1-delNT26-181_              NGEVQAEIRK SWSRWTLALD FKRKARSGSS SYSYGPMVSH TSVTNVGPRA
hPTHR1-delNT-6E                  NGEVQAEIKK SWSRWTLALD FKRKARSGSS SYSYGPMVSH TSVTNVGPRV
hPTHR1_6E                        NGEVQAEIKK SWSRWTLALD FKRKARSGSS SYSYGPMVSH TSVTNVGPRV
hPTHR1_delNT-YFP                 NGEVQAEIKK SWSRWTLALD FKRKARSGSS SYSYGPMVSH TSVTNVGPRV

                                 751                                                    800
hPTHR1                           GLGLPLSPRL LPTATTNGHP QLPGHAKPGT PALETLETTP PAMAAPKDDG
hPTHR1-GFP                       GLGLPLSPRL LPTATTNGHP QLPGHAKPGT PALETLETTP PAMAAPKDDG
rPTHR1-delNT26-181_              GLSLPLSPRL P.PATTNGHS QLPGHAKPGA PATET.ETLP VTMAVPKDDG
hPTHR1-delNT-6E                  GLGLPLSPRL LPTATTNGHP QLPGHAKPGT PALETLETTP PAMAAPKDDG
hPTHR1_6E                        GLGLPLSPRL LPTATTNGHP QLPGHAKPGT PALETLETTP PAMAAPKDDG
hPTHR1_delNT-YFP                 GLGLPLSPRL LPTATTNGHP QLPGHAKPGT PALETLETTP PAMAAPKDDG


                                 801                                 832
hPTHR1                           FLNGSCSGLD EEASGPERPP ALLQEEWETV M*
hPTHR1-GFP                       FLNGSCSGLD EEASGPERPP ALLQEEWETV M*
rPTHR1-delNT26-181_              FLNGSCSGLD EEASGSARPP PLLQEGWETV M*
hPTHR1-delNT-6E                  FLNGSCSGLD EEASGPERPP ALLQEEWETV M*
hPTHR1_6E                        FLNGSCSGLD EEASGPERPP ALLQEEWETV M*
hPTHR1_delNT-YFP                  FLNGSCSGLD EEASGPERPP ALLQEEWETV M*
```

Supplementary Figure 19. Annotated sequence data for PTHR1 constructs. Alignment was performed using ClustalOmega. The sequence from exon 2 is underlined. Constructs for hPTHR1 GFP[2], rat PTHR1 lacking extracellular domain residues 26-181 (rPTHR1-delNT26-181)[3], human PTHR1 lacking extracellular domain residues 26-181 with 6E tag incorporated (hPTHR1-delNT-6E) and human PTHR1 lacking extracellular domain residues 26-181 with YFP incorporated (hPTHR1-delNT-YFP) are shown here. The GFP insert is shown in green, the YFP insert in gold, and the 6E tag in purple. Residues 1-22 in hPTHR1 correspond to the signal peptide.

```
VHH-PTHR  1    EVQLVESGGGLVQAGGSLRLSCAASGNIFANNIMGWYRQPPGKEREFVAHVSHDGDSMyA--VSVKGRFAISRKDA-TNL  77
VHH-6E    1    QVQLQESGGGLVQPGGSLRLSCAASGFVFENSAMAWYRQAPGKERELIAVIGTTFIKL-A--ESVKGRFTISRDNAKSTV  77
VHH-GFP   1    QVQLQESGGALVQPGGSLRLSCAASGFPVNRYSMRWYRQAPGKEREWVAGMSSAGDRS-SYEDSVKGRFTISRDDARNTV  79
VHH-Kappa 1    QVQLVESGGGWVQPGGSLRLSCAASGFTFSDTAMMWVRQAPGKGREWVAAIDTGGGYT-YYADSVKGRFTISRDNAKNTL  79

VHH-PTHR       YLQMNSLKPEDTAIYFCrllniptqGRMEG--FW-----------GQGTQVTVSS--LPETGGHHHHHH  131
VHH-6E         YLQMNNLKPEDTAVYYC--------SKSGA--YW-----------GQGTQVTVSSGGLPETGGHHHHHH  125
VHH-GFP        YLQMNSLKPEDTAVYYC--------NVNVGFEYW-----------GQGTQVTVSSGGLPETGGHHHHHH  129
VHH-Kappa      YLQMNSLKPEDTARYYC--------AKTYSGNYYsnytvanygttGRGTLVTVSSGGLPETGGHHHHHH  140
```

Supplementary Figure 20. Alignment of VHHs used in this study. Red lettering indicates complete conservation. Blue lettering indicates partial conservation. Black lettering indicates no conservation. Sequences were aligned using Cobalt (Constraint-based Multiple Alignment Tool). The sequence GGLPETGGHHHHHH is not part of the VHH and was cloned in for sortagging.

	hPTHR1			hPTHR1$_{GFP}$			hPTHR1$_{6E}$		
	EC$_{50}$ (nM ± SD)	Max (normalized ± SD)	*n*	EC$_{50}$ (nM ± SD)	Max (normalized ± SD)	*n*	EC$_{50}$ (nM ± SD)	Max (normalized ± SD)	*n*
PTH(1-34)	0.51 ± 0.28	1.00 ± 0.00	8	0.4 ± 0.8	1.00 ± 0.00	7	1.3 ± 1.0	1 ± 0.0	3
PTH(1-14)	4.3 ± 2.0	1.01 ± 0.07	5	2.3 ± 1.2	1.09 ± 0.03	3	3.4 ± 1.6	1.06 ± 0.12	3
PTH(1-11)	516 ± 238	1.01 ± 0.09	7	79 ± 45	1.10 ± 0.13	7	94 ± 74	1.09 ± 0.22	3
PTH(1-10)	3121 ± 1671	1.03 ± 0.08	3	2552 ± 653	1.16 ± 0.15	6	5079 ± 407	1.09 ± 0.07	3
PTH(1-9)	Inactive at 10,000 nM		3	Inactive at 10,000 nM		4	Inactive at 10,000 nM		3
VHH$_{PTHR}$-PTH(1-14)	0.075 ± 0.041	0.96 ± 0.17	4	1.7 ± 2.3	1.10 ± 0.10	3	0.2 ± 0.1	0.98 ± 0.19	3
VHH$_{PTHR}$-PTH(1-11)	5.0 ± 1.6	0.95 ± 0.09	7	0.5 ± 0.1	1.03 ± 0.26	4	4.0 ± 3.2	0.98 ± 0.11	3
VHH$_{PTHR}$-PTH(1-10)	Inactive at 100 nM		2	ND			ND		
VHH$_{6E}$-PTH(1-14)	Inactive at 330 nM		3	32.9 ND 3.5	1.14 ± 0.08	3	0.4 ± 0.2	1.08 ± 0.23	3
VHH$_{6E}$-PTH(1-11)	Inactive at 100 nM		2	Inactive at 100 nM		3	6.9 ± 2.6	1.11 ± 0.18	3
VHH$_{6E}$-PTH(1-10)	ND			Inactive at 100 nM		3	2.8 ± 1.4	0.94 ± 0.04	3
VHH$_{6E}$-PTH(1-9)	ND			ND			Inactive at 100 nM		3
VHH$_{GFP}$-PTH(1-14)	Inactive at 100 nM		3	1.8 ± 0.6	1.21 ± 0.17	3	Inactive at 100 nM		2
VHH$_{GFP}$-PTH(1-11)	Inactive at 100 nM		3	8.0 ± 3.4	1.10 ± 0.18	6	Inactive at 100 nM		2
VHH$_{GFP}$-PTH(1-10)	ND			Inactive at 100 nM		3	ND		
VHH$_{GFP}$-PTH(1-9)	ND			Inactive at 100 nM		4	ND		

	hPTHR1$_{YFP\Delta ECD}$			rPTHR1			hPTHR2		
	EC$_{50}$ (nM ± SD)	Max (normalized ± SD)	*n*	EC$_{50}$ (nM ± SD)	Max (normalized ± SD)	*n*	EC$_{50}$ (nM ± SD)	Max (normalized ± SD)	*n*
PTH(1-34)	689 ± 301	1.00 ± 0.00	7	0.28 ± 0.12	1.00 ± 0.00	3	1.45 ± 2.42	1 ± 0	4
PTH(1-14)	1.1 ± 0.9	0.96 ± 0.04	4				924 ± 328	1.14 ± 0.28	3
PTH(1-11)	246 ± 133	1.02 ± 0.12	10	120.3 ± 90.3	1.02 ± 0.03	3	>100,000 (20% at 100 uM)	0.36 ± 0.32	3
PTH(1-10)	3841 ± 1604	1.12 ± 0.08	6	2466 ± 23	1.09 ± 0.11	3			
PTH(1-9)	Inactive at 10,000 nM		3						
VHH$_{PTHR}$-PTH(1-14)	0.9 ± 0.5	0.91 ± 0.15	4				Inactive at 330 nM		3
VHH$_{PTHR}$-PTH(1-11)	Inactive at 100 nM		3	3.2 ± 0.9	1.00 ± 0.13	3			
VHH$_{PTHR}$-PTH(1-10)	ND								
VHH$_{6E}$-PTH(1-14)	72 ± 15.2	1.11 ± 0.07	3				Inactive at 330 nM		3
VHH$_{6E}$-PTH(1-11)	Inactive at 100 nM		3	Inactive at 100 nM		2			
VHH$_{6E}$-PTH(1-10)	ND								
VHH$_{6E}$-PTH(1-9)	ND								
VHH$_{GFP}$-PTH(1-14)	0.58 ± 0.29	1.16 ± 0.36	4						
VHH$_{GFP}$-PTH(1-11)	0.14 ± 0.06	1.19 ± 0.40	7	Inactive at 100 nM		2			
VHH$_{GFP}$-PTH(1-10)	0.46 ± 0.22	1.06 ± 0.35	7						
VHH$_{GFP}$-PTH(1-9)	~40% activation at 100 nM	0.38 ± 0.11	5						

Supplementary Table 1. Complete tabulation of cAMP induction assays. Data are identical to those presented in Table 1 and Supplementary Figure 17. Maximal responses (Max) were normalized relative to the top plateau value induced by saturating values of PTH(1-34) for all receptors besides hPTHR1$_{YFP\Delta ECD}$, for which PTH(1-11) was used.

References.

1 Wehbi, V. L. *et al.* Noncanonical GPCR signaling arising from a PTH receptor-arrestin-G beta gamma complex. *Proceedings of the National Academy of Sciences of the United States of America* **110**, 1530-1535, doi:10.1073/pnas.1205756110 (2013).

2 Mahon, M. J. pHluorin2: an enhanced, ratiometric, pH-sensitive green florescent protein. *Adv Biosci Biotechnol* **2**, 132-137, doi:10.4236/abb.2011.23021 (2011).

3 Lee, C. W. *et al.* Role of the extracellular regions of the parathyroid-hormone (PTH) PTH-related peptide receptor in hormone-binding. *Endocrinology* **135**, 1488-1495, doi:10.1210/en.135.4.1488 (1994).
