# Supplementary material for: Improved GPCR ligands from nanobody tethering
Source: Nat Commun. 2020 Apr 29;11:2087. doi: 10.1038/s41467-020-15884-8 (PMC7190724; doi:10.1038/s41467-020-15884-8)
Supplement: Supplementary file 2 — Reporting Summary [file 41467_2020_15884_MOESM2_ESM.pdf]

nature research

Corresponding author(s): Thomas Gardella
Hidde Ploegh

Last updated by author(s): 27-Mar-2020

Reporting Summary

Nature Research wishes to improve the reproducibility of the work that we publish. This form provides structure for consistency and transparency in reporting. For further information on Nature Research policies, see Authors & Referees and the Editorial Policy Checklist .

Statistics

For all statistical analyses, confirm that the following items are present in the figure legend, table legend, main text, or Methods section.

n/a	Confirmed	
[ ]	[x]	The exact sample size (*n*) for each experimental group/condition, given as a discrete number and unit of measurement
[ ]	[x]	A statement on whether measurements were taken from distinct samples or whether the same sample was measured repeatedly
[ ]	[x]	The statistical test(s) used AND whether they are one- or two-sided<br>*Only common tests should be described solely by name; describe more complex techniques in the Methods section.*
[x]	[ ]	A description of all covariates tested
[ ]	[x]	A description of any assumptions or corrections, such as tests of normality and adjustment for multiple comparisons
[ ]	[x]	A full description of the statistical parameters including central tendency (e.g. means) or other basic estimates (e.g. regression coefficient) AND variation (e.g. standard deviation) or associated estimates of uncertainty (e.g. confidence intervals)
[ ]	[x]	For null hypothesis testing, the test statistic (e.g. *F*, *t*, *r*) with confidence intervals, effect sizes, degrees of freedom and *P* value noted<br>*Give P values as exact values whenever suitable.*
[x]	[ ]	For Bayesian analysis, information on the choice of priors and Markov chain Monte Carlo settings
[x]	[ ]	For hierarchical and complex designs, identification of the appropriate level for tests and full reporting of outcomes
[x]	[ ]	Estimates of effect sizes (e.g. Cohen's *d*, Pearson's *r*), indicating how they were calculated

Our web collection on statistics for biologists contains articles on many of the points above.

Software and code

Policy information about availability of computer code

Data collection	Data from plate-based assays was collected using SoftMaxPro version 6.2.1. Data from mass spectrometry experiments was collected using MassLynx version 4.1. Flow cytometry data was collected using BD cSampler software. Blood calcium levels were collected using software on Siemens RapidLab 348 Ca2+/pH analyzer.
Data analysis	Data was analyzed using GraphPad Prism 6 or Microsoft Excel 2016.

For manuscripts utilizing custom algorithms or software that are central to the research but not yet described in published literature, software must be made available to editors/reviewers. We strongly encourage code deposition in a community repository (e.g. GitHub). See the Nature Research guidelines for submitting code & software for further information.

Data

Policy information about availability of data

All manuscripts must include a data availability statement. This statement should provide the following information, where applicable:
- Accession codes, unique identifiers, or web links for publicly available datasets
- A list of figures that have associated raw data
- A description of any restrictions on data availability

The data that support the findings of this study are available from the corresponding authors upon reasonable request.

Field-specific reporting

Please select the one below that is the best fit for your research. If you are not sure, read the appropriate sections before making your selection.

Life sciences study design

All studies must disclose on these points even when the disclosure is negative.

Sample size	No sample size calculations were performed. Replicates were performed in line with previous studies of this type.
Data exclusions	No data was excluded
Replication	Data were confirmed using independent experiments carried out the same laboratory as indicated in figure captions. Note that Figure 5 was not replicated.
Randomization	Mice were grouped so that basal blood calcium levels (prior to injections) were not statistically different.
Blinding	Blinding was not performed as the variable measured (blood calcium) was quantified by an automated machine and was not subject to investigator input. Mice were not handled beyond blood draws during the course of the experiment.

Reporting for specific materials, systems and methods

We require information from authors about some types of materials, experimental systems and methods used in many studies. Here, indicate whether each material, system or method listed is relevant to your study. If you are not sure if a list item applies to your research, read the appropriate section before selecting a response.

Materials & experimental systems

n/a	Involved in the study	
☐	☒	Antibodies
☐	☒	Eukaryotic cell lines
☒	☐	Palaeontology
☐	☒	Animals and other organisms
☒	☐	Human research participants
☒	☐	Clinical data

Methods

n/a	Involved in the study	
☒	☐	ChIP-seq
☐	☒	Flow cytometry
☒	☐	MRI-based neuroimaging

Antibodies

Antibodies used	The source of each of the nanobodies used in the study is referenced. The anti-HA commercial antibody is described in the methods section.
Validation	Nanobodies were validated using negative control cell lines, lacking only the antigen of interest, ensuring any positive signal is due to the expression of the antigen.

Eukaryotic cell lines

Policy information about cell lines

Cell line source(s)	HEK293 cells were originally purchased from ATCC. Clonal cell lines expressing GloSensor, PTHR1 and variants were produced in the investigators laboratory (TG).
Authentication	Cell lines were tested for their ability to respond to PTH and produce luminescence (from Glosensor expression)
Mycoplasma contamination	Cell tested negative for mycoplasma contamination
Commonly misidentified lines (See ICLAC register)	None

Animals and other organisms

Policy information about studies involving animals; ARRIVE guidelines recommended for reporting animal research

Laboratory animals	Mice (CD1 female, age 11 weeks)
Wild animals	None used
Field-collected samples	None used

Ethics oversight	Mice were treated in accordance with the ethical guidelines adopted by Massachusetts General Hospital

Note that full information on the approval of the study protocol must also be provided in the manuscript.

Flow Cytometry

Plots

Confirm that:

- [x] The axis labels state the marker and fluorochrome used (e.g. CD4-FITC).
- [x] The axis scales are clearly visible. Include numbers along axes only for bottom left plot of group (a 'group' is an analysis of identical markers).
- [x] All plots are contour plots with outliers or pseudocolor plots.
- [x] A numerical value for number of cells or percentage (with statistics) is provided.

Methodology

Sample preparation	Suspensions of cells in PBS were stained for 1 hour on ice in the presence of indicated concentrations of VHH probes functionalized with Alexafluor647. Cells were pelleted by centrifugation and washed with PBS prior to analysis by flow cytometry
Instrument	(BD Accuri C6).
Software	Data was analyzed using FlowJo version 7.6.
Cell population abundance	Not analyzed.
Gating strategy	Gating was performed on forward scatter/side scatter profiles to analyze intact cells.

- [x] Tick this box to confirm that a figure exemplifying the gating strategy is provided in the Supplementary Information.
